# Perspectives on self-managed abortion among providers in hospitals along the Texas–Mexico border

**DOI:** 10.1186/s12905-021-01281-w

**Published:** 2021-03-30

**Authors:** Sarah Raifman, Sarah E. Baum, Kari White, Kristine Hopkins, Tony Ogburn, Daniel Grossman

**Affiliations:** 1 Advancing New Standards in Reproductive Health (ANSIRH), Department of Obstetrics, Gynecology & Reproductive Sciences, University of California, San Francisco, 1330 Broadway, Suite 1100, Oakland, CA 94612 USA; 2 Ibis Reproductive Health, 1736 Franklin St, Suite 600, Oakland, CA 94612 USA; 3 Steve Hicks School of Social Work, The University of Texas at Austin, 1925 San Jacinto Blvd, Stop D3500, TX 78712 Austin, USA; 4 Population Research Center, The University of Texas at Austin, 305 E. 23rd St. Stop G1800, Austin, TX 78712-1699 USA; 5 University of Texas Rio Grande Valley, 2102 Treasure Hill Blvd, Harlingen, TX 78550 USA

**Keywords:** Abortion, Pregnancy intention, Qualitative research methods, Service providers, United States

## Abstract

**Background:**

Following self-managed abortion (SMA), or a pregnancy termination attempt outside of the formal health system, some patients may seek care in an emergency department. Information about provider experiences treating these patients in hospital settings on the Texas-Mexico border is lacking.

**Methods:**

The study team conducted semi-structured interviews with physicians, advanced practice clinicians, and nurses who had experience with patients presenting with early pregnancy complications in emergency and/or labor and delivery departments in five hospitals near the Texas-Mexico border. Interview questions focused on respondents’ roles at the hospital, knowledge of abortion services and laws, perspectives on SMA trends, experiences treating patients presenting after SMA, and potential gaps in training related to abortion. Researchers conducted interviews in person between October 2017 and January 2018, and analyzed transcripts using a thematic analysis approach.

**Results:**

Most of the 54 participants interviewed said that the care provided to SMA patients was, and should be, the same as for patients presenting after miscarriage. The majority had treated a patient they suspected or confirmed had attempted SMA; typically, these cases required only expectant management and confirmation of pregnancy termination, or treatment for incomplete abortion. In rare cases, further clinical intervention was required. Many providers lacked clinical and legal knowledge about abortion, including local resources available.

**Conclusions:**

Treatment provided to SMA patients is similar to that provided to patients presenting after early pregnancy loss. Lack of provider knowledge about abortion and SMA, despite their involvement with SMA patients, highlights a need for improved training.

## Introduction

Growing evidence suggests that some people in the United States (US) manage the termination of a pregnancy on their own outside of the health care system [1–6]. Patients who attempt self-managed abortion (SMA) often cite barriers to facility-based care as a motivating factor [1], and the practice may be more common in states where access to abortion is highly restricted, such as Texas [2, 3]. A study of Texas abortion patients in McAllen, San Antonio, and Fort Worth conducted in 2017–2018 found that 16%, 9%, and 15%, respectively, reported seeking or trying any method to end their pregnancy before going to the clinic [2]. Patients reported using the following SMA methods: herbs, medications including misoprostol, and homeopathic remedies [2]. In addition, evidence suggests that the number of online requests for mifepristone and misoprostol increased when clinics were shut down early in the COVID-19 pandemic [4]. SMA prevalence estimates are higher in Texas relative to a national 2014 study, which found that approximately 2% of US abortion patients have ever attempted SMA [5, 6]. The legal context around SMA is evolving in the US: as of 2019, there are roughly 40 laws that potentially could be used to prosecute someone who self manages an abortion with the support of an advocate or caregiver, and there have been 21 known arrests for SMA and criminal investigations in 20 states, including Texas [7]. Six states directly ban SMA as of 2019 (Idaho, Nevada, Arizona, Oklahoma, South Carolina, and Delaware) [8].

A variety of factors may contribute to SMA being more common in Texas, and specifically in the Texas-Mexico border region, relative to other US states. These include geographic proximity to Mexico where medications are generally inexpensive and less regulated than in the US [9]; the availability of misoprostol, a medication used for termination of pregnancy, without a prescription in Mexican pharmacies [10]; the large population of immigrants from Latin America, where people have been using misoprostol for SMA in contexts where abortion is legally restricted [11]; and the limited availability of facility-based abortion care in Texas. A qualitative study of Texas women with SMA experience found that those who used misoprostol had obtained it in Mexico [4]. Among other legal restrictions on abortion patients, providers, and facilities, Texas implemented House Bill 2, one of the most restrictive abortion laws in the country, in 2013, which led to the closure of half the state’s abortion clinics [2, 12]. Perceived and experienced abortion stigma among people seeking abortion in Texas, due to religious, cultural and political influences, also likely leads some to seek abortion outside of the formal medical system. A desire to avoid stigma or shame, in addition to recommendations from friends or family members, are reported as reasons for attempting SMA in Texas [4]. A survey of Texas women age 18–49 found that 22% reported knowing someone who had attempted SMA; those more likely to report knowing someone who attempted SMA included Latinas living in a county bordering Mexico and people reporting they faced financial or logistical barriers accessing reproductive health services [13].

Historically, clinicians working in emergency departments (EDs) have had limited exposure to patients seeking care after a facility-based abortion. This is in part because complications are rare after abortion: one study in California of over 500,000 abortion patients in 2009–2010 found that 0.9% visited an ED for an abortion-related complication within 6 weeks of the abortion [14]. A national study found that 0.01% of all ED visits by women aged 15–49 in 2009–2013 were abortion-related, of which 51% required only observation without treatment [15]. Patients who travel farther to access abortion services are more likely to seek care in an ED in case of a possible complication after returning home, suggesting that as facility-based abortion care becomes more difficult to access—or even non-existent in some states—more patients may present to EDs for abortion-related care [16].

National data suggest that ED visits related to SMA in particular are even less common, representing 1.4% of abortion-related ED visits. Higher rates of SMA-related ED visits were documented in the South (2%) compared to the Midwest (1%), West (1.1%), and Northeast (1.3%) [15]. There are research gaps in our understanding of the provision of SMA-related treatment in the hospital setting. Studies documenting significant barriers to providing miscarriage management in the ED suggest that SMA care may be similarly challenging [17]. Given the additional risk of criminalization faced by patients suspected of attempting SMA, the limited experience ED providers historically have had managing these patients, and the changing policy environment that may lead to an increase in SMA, it is critical to explore the perspective of clinicians who may care for these patients in urgent care settings. For some who try SMA, ED providers may be the only point of contact with the medical system; therefore, ED providers are well-positioned to provide insight into SMA trends and experiences of patients who seek hospital care, which could inform guidance to improve their care.

The aim of this exploratory study was to document hospital provider perspectives of SMA, the care provided to patients who present to the ED after attempting SMA, and provider preparedness to respond to these patients. We explored these questions through interviews with providers who had experience treating patients who presented with early pregnancy complications in hospital settings, including the ED and labor and delivery (L&D), to document their experiences with patients presenting after possible SMA. ED and L&D provider perspectives can offer new insight into SMA trends and the needs of patients and providers as facility-based abortion care becomes increasingly more restricted in Texas.

## Methodology

To capture a diversity of perspectives among providers with experience treating patients with early pregnancy complications, we recruited and interviewed physicians, advance practice clinicians (APCs), and nurses who worked in the ED and/or L&D at five hospitals in Texas near the Mexican border in October 2017 and January 2018. We chose this study population to enable the investigation of not only experience managing SMA and suspected SMA, but also gaps in preparedness, training, and resources available to providers who treat patients presenting after potential SMA. We identified recruitment hospitals along the border based on interest in the study among providers and administrators across the region. These contacts assisted us in obtaining institutional approval for the study and in identifying eligible participants. We included providers working in L&D settings after learning early in the study that some cases of suspected SMA—particularly those later in gestation—were sometimes managed in the obstetrical unit, including L&D or an obstetrics triage, rather than the ED setting.

Initial efforts to remotely recruit participants for phone interviews using departmental announcements and email listservs were not effective. We conducted two pilot interviews by telephone, after which we edited the interview guide to improve clarity and add probing questions related to key themes. We excluded these pilot interviews from analysis. In our revised recruitment strategy, three researchers trained in qualitative data collection visited sites to recruit and interview participants in person. At each hospital, the primary contact initially informed their colleagues about the study and invited them to go to a private room where the researchers were conducting interviews to schedule an interview if they were interested in participating. We also employed a snowball sampling approach: after participants completed their interview, we encouraged them to inform eligible colleagues about the study. The study did not provide incentives for participation.

Providers were eligible to participate if they worked at any of the five study hospitals and had experience with patients presenting with early pregnancy complications in the ED or L&D. Eligible participants provided informed consent to conduct and audio-record the interview. At each site, we conducted as many interviews as possible over the course of two or three days. We aimed to complete 50 interviews with perspectives from five hospitals. We conducted the research in accordance with the relevant guidelines and regulations after obtaining ethical approval from institutional review boards at the University of California, San Francisco and The University of Texas at Austin and official administrative approval from each hospital site.

As one of the first studies to explore this topic with ED and L&D providers, it was exploratory. The interview guide and analysis were grounded in the existing literature around SMA and miscarriage management [4, 17–19]. Semi-structured interview questions focused on the respondent’s clinical experience, role at the hospital, primary patient population, knowledge of local abortion services and relevant state laws, perspective on trends in SMA, experience with care provided to patients presenting after a potential SMA attempt, and perceived gaps in training related to SMA. Interviewers also asked participants about specific cases in which patients had attempted SMA, including the patient’s clinical presentation, weeks’ gestation, methods used, treatment, outcome and complications, as well as the challenges the provider may have faced treating the patient, lessons learned about the experience, and personal feelings about SMA and/or abortion. The length of interviews varied to accommodate hospital flow and providers’ time constraints. For providers who had limited time, the interviewers prioritized key topics in the interview guide including SMA trends and experience treating SMA patients. The research team also collected minimal demographic information from providers at the end of the interviews, including their age, gender, race, and ethnicity.

The interviewers reviewed the professionally transcribed interviews to ensure consistency with the recalled encounter and the audio recording as needed and to redact any identifying information. The research team developed a preliminary codebook based on recurring patterns they identified during the interview process. Using Dedoose [20], three researchers trained in qualitative coding and analysis independently coded a subset of interviews using the preliminary codebook and then compared their respective applications of the codes, discussed inconsistencies, resolved differences through consensus, refined the codebook, and applied the revised codebook to a new subset of transcripts. This process was repeated three times until the researchers agreed on a final codebook. The coders met regularly to discuss any new themes that emerged and, through consensus, added them to the codebook. Once there was concordance, two coders independently applied the final codebook to all transcripts. The first author then used a matrix to analyze patterns in the data [21] and repeatedly revisited the transcripts to ensure that codes were applied to all relevant text and that all key themes were included in the matrix. Below, we summarize major themes and identify participants by their respective roles (physician, APC, or nurse) and departments (ED or L&D) at the time of the interview.

## Results

We completed 57 interviews at five hospitals and omitted three incomplete interviews (defined as shorter than ten minutes), which did not include relevant experience with SMA. The final analysis included 54 interviews between ten minutes and one hour in length. One-third of respondents in the sample were physicians (n = 18) and two-thirds served in other clinical roles, primarily as nurses (Table 1). Nearly two-thirds held positions in L&D and one-third in the ED, though some providers served in both departments. The type of providers interviewed was not equally distributed across hospital sites, likely due to use of snowball recruitment methods: two of the sites included only physicians or only nurses. The majority of participants self-identified as female (n = 37, 69%) and Hispanic (n = 35, 65%). One-third of providers were in their 30s (n = 18, 33%), with the remainder split between their 20s (n = 12, 22%), 40s (n = 11, 20%), and 50 years or older (n = 10, 19%).

**Table 1:** Participant characteristics

Participant characteristics (n = 54)	
Clinical role	n (%)
Physician	18 (33%)
Advanced Practice Clinician (APC) (nurse practitioner, physician’s assistant)	4 (8%)
Nurse (Registered nurse, licensed vocational nurse)	32 (59%)
Department	
Emergency	20 (37%)
Labor and delivery	34 (63%)
Sex	
Female	37 (69%)
Male	17 (32%)
Age	
< 30 years	12 (22%)
30–39 years	18 (33%)
40–49 years	11 (20%)
≥ 50 years	10 (19%)
Missing	3 (6%)
Hispanic/Latin origin	
Yes	35 (65%)
No	14 (26%)
Missing	5 (9%)
Number of SMA cases discussed	
0	16 (30%)
1	27 (50%)
2	10 (19%)
3	1 (2%)
Facility	
Hospital 1	13 (24%)
Hospital 2	8 (15%)
Hospital 3	15 (28%)
Hospital 4	10 (19%)
Hospital 5	8 (15%)

Proportions may not add up to 100% as a result of rounding

We describe four main themes that emerged: trends in SMA patient characteristics and trends; treatment protocols for suspected SMA patients; involvement of social workers or police in suspected SMA cases; and provider knowledge about abortion and gaps in training for treatment of SMA patients.

### Provider perception of SMA patient characteristics and trends

In general, respondents indicated that most patients they suspected of SMA were early in gestation, young, presenting for care on their own, and had minimal resources, including no health insurance. An ED physician noted, “Well they’re usually in the early first trimester. Anywhere between four and eight weeks I would say on average. And they present with bleeding and cramping. …they’re usually young. But not always. I mean they’re typically between 18 and 24 or younger…. Although I have seen a couple of patients that were older than that. They usually show up alone.”

Another physician who worked on L&D recalled that many of the SMA patients seen at the hospital were originally from Mexico: “Most of them were undocumented, but had formed a life over here in [city], but for certain issues, they couldn’t establish care, monetary issues to get the abortion, so they went to Mexico or had a family member go to Mexico and obtain some medication for them.”

Providers’ anecdotal estimates of the frequency of seeing patients suspected of SMA in the hospital ranged from one case per month to 10 cases in 20 years. The majority of providers reported first-hand experience treating patients who attempted SMA, while others had only heard about SMA happening in the community. Of the 54 respondents, 38 (70%) recalled at least one case of suspected SMA in which they were personally involved.

Providers reported a range of responses regarding SMA trends in the community. At one facility, two respondents perceived recent increases in SMA; an ED nurse said: “I think that we have more [SMA] cases now. I think that part of it might have been the fact that we don’t—it’s harder to get access to abortion here under a doctor’s care, as opposed to what it was before…I think that may be part of it.” An ED physician who worked in the same hospital also noted “seeing an upsurge” in SMA cases. When asked about when this change occurred, they said: “…since the internet provided information about what Cytotec is, how available it is, and because we are a border area it’s fairly available and most patients will have a grandmother who’s on Cytotec for ulcer treatment or GI discomfort, or that sort of thing. It’s relatively cheap.”

In contrast, two providers who had been working in the region for over two decades each said they thought SMA was less common now than it was in the past. One of these providers, an L&D nurse, explained: “I haven’t seen it since I’ve been back [to the region] this time. I used to work here in 1994,’95,’96, and we had several women that came in after they had taken Cytotec because you can get that in Mexico.”

### Treatment protocols for suspected SMA patients

Overall, providers reported that they were clinically prepared to treat SMA patients primarily because SMA treatment requires the same knowledge and skills as miscarriage management. Respondents described SMA patients as having similar presentation as those experiencing early miscarriage and then receiving either treatment for incomplete abortion (typically vacuum aspiration) or expectant management (observation without treatment) at the ED and/or L&D. An L&D physician explained a common presentation: “Typically [patients] come in because they started the process, maybe they don’t realize what it entails, so then they kind of get worried because they start cramping and bleeding a lot. So they’ll come to the ED. So, a lot of times it’s just reassurance that, you know, what they’ve done—this is just part of the process.”

Most participants said they had not witnessed any complications related to SMA; however, a handful reported cases that required additional treatment, such as dilation and curettage for retained placenta or a blood transfusion, or monitoring of their continuing pregnancy after attempting SMA. Respondents indicated that the type of cases they saw in the hospital were likely not representative of typical SMA experiences. For example, an L&D physician said, “We've only seen the grave complications. I'm sure there's a lot of patients that successfully do self-induce with misoprostol from Mexico that we don't even hear about.” An ED nurse at a different hospital agreed with this sentiment, “The few we do see are the ones that weren’t lucky, and they ended up sick because of it.”

When asked about the type of care provided to patients presenting after a possible SMA, the majority of participants explained that it was no different from the care provided to any pregnant patient presenting with bleeding or signs of early pregnancy complications. Most providers said they did not typically inquire about pregnancy intention or about whether a patient had done something to try to end the pregnancy on their own, and if they did, it did not affect their clinical decision-making. An L&D nurse said, “We’re nurses here, we’re going to care for them whether they wanted to keep their baby or really lost their baby or really didn’t want to have nothing to do with the baby. Our care is the same.” This was echoed by other nurses. Another L&D nurse at the same hospital said: “they’re not really treated differently than someone who comes with a spontaneous miscarriage.” An ED nurse at a different hospital said: “It doesn’t change anything. I was like, if you took the pills, you didn’t take the pills, who cares? Like, we’re going to medically screen you, treat you, and then send you on your way. Or admit you if you need to be admitted. [Our care] has nothing to do [with whether] you took pills or not.”

Some providers reported having asked about whether a patient had done something to terminate the pregnancy, but that asking was by no means part of a standard protocol. Instead, providers referenced noticing something that made them ask about a patient’s potential SMA attempt or pregnancy intention. For example, an L&D nurse explained, “…you notice things, discrepancies in their stories, and you try to dig a little bit more… that’s when you start catching on that something’s not right with this story…but once they get that information out, we don’t do much else.” The majority of those who said they ask about pregnancy intention indicated that their motivation was to better understand the patient’s social situation in order to refer them as needed to additional services; none said that the information was necessary to provide better clinical care for the patient.

### Reflections on reporting suspected SMA cases

In addition to providing the same care to all patients presenting with pregnancy complications, regardless of SMA attempts, more than half of the providers also felt that there was no need to report suspected SMA cases to the police or other authority. None of the respondents knew of policies or guidelines for reporting specific to suspected SMA, and most said that the decision to report such cases was subject to provider discretion. An ED physician said: “To my knowledge, there's no reporting, per se, that needs to be done. From the medical/legal standpoint there's… I mean, like I said, lawfully, termination of pregnancy is a lawful act. So I don't see any reporting that needs to be reported. And, to my knowledge, there is no law that says we have to, you know, call any state agency, etcetera.” An L&D nurse explained, “It’s kind of either the provider or the nurse [who] makes that decision…a lot of times, [patients] won’t tell the provider certain things, and they’ll mention things to us, and we’re like, okay, we need to find out about that.” This provider continued on to offer some examples of when they might call social services to further support the patient, including if the patient has suicidal tendencies or problems at home, such as lack of supplies or resources to care for the baby should the pregnancy continue.

Several respondents recalled a suspected SMA case where social workers were asked to participate in the patient’s care, which sometimes led to the involvement of Child Protective Services (CPS) or the police. Two broader categories of circumstances led to the involvement o social workers: protecting patients against possible coercion or abuse and reacting to potential harm to a viable fetus. This comment from an L&D nurse illustrates the first category, “If the person is a minor or homeless or maybe doesn’t have transportation, any kind of abuse issue…[social work may become involved] to see if there’s any issues that will present to the woman when she gets home.” Another L&D nurse at a different hospital discussed involving social workers in the case of a patient from Central America who presented at 20 weeks’ gestation after having taken pills multiple times to terminate the pregnancy. When asked for the primary reason social workers were called, the nurse said: “Because we found out it wasn’t the first time that she had taken the pills…we started trying to dig and find out ‘hey, what’s going on? And why would you want to?’ you never know; it could have been a rape here? If it was a rape that happened to them as they were traveling, there’s not a lot we can do, but if it’s something that’s going on here? Then we can escalate that and try to get our authorities, or whatever it is we need to do.” Sometimes concern about a viable fetus was used to justify the involvement of social workers, particularly if the patient was beyond the first trimester in pregnancy. One L&D physician likened the situation to drug use in pregnancy, suggesting that SMA attempts after 20 weeks’ gestation justified the involvement of social workers.

Two of the reported cases in which the police were notified involved patients who were minors. An ED nurse, who was working in the unit at the time the patient presented but was not directly involved in the case, believed the police were called because the minor had reportedly purchased medications used to terminate the pregnancy at school. The provider said: “We had to call case management. Case management has to inform police. And she gave the name of her friend who gave it, and they had to go to the school and talk to her also. They also had to talk to the mother. I think in that case, because it involved a school—it came from the school—so I think that's why they had to call the police to let them know, so she won’t bring more to the school and give it to, you know, other kids.”

In the case of another minor, the provider involved the police because of possible partner coercion. The patient had attempted SMA unsuccessfully throughout her pregnancy and was seen again in the ED at 31 weeks’ gestation after taking pills she had purchased in Mexico. She was accompanied by her boyfriend, who was married, and the providers had the impression that it was he, not she, who did not want the pregnancy to continue. The provider, an ED nurse, explained: “In this case, CPS just got involved because of the abuse that [her partner] had made her do it, but she was not willing to file any charges against him. So, the police officer could not hold him. CPS only gets involved usually if there's any kind of abuse or if she were to have any issue to come back positive for any drugs, which she was negative for.”

A minority of providers said they were against abortion in general and that they would be in favor of reporting patients who attempted SMA, particularly at later gestations. One of these was an ED nurse who explained, “I would definitely like to see the policy change, that if [the fetus is] viable that it’s against the law, and that there be some criminal back-up for the patient to where something happened to her.”

### Training needs related to SMA and abortion

As discussed above, nearly all providers reported being clinically equipped to manage the treatment of patients presenting with pregnancy loss and associated complications, including related to SMA. However, several key gaps in preparedness for responding to the needs of SMA patients remained. Several providers, mainly nurses, revealed a lack of basic knowledge about the safety, side effects, and potential complications of the medication abortion process. For example, an ED nurse said, “I don’t think [patients] understand the severity of that pill. The side effects… I can’t remember what it is…bleeding and pain and nausea and vomiting… the overnight pill to abort.” Another ED nurse acknowledged their own lack of information about medication abortion, “…I don’t know what [the abortion pill] is called. I think it’s like… it’s just a more intense, or higher dosage of a Plan B, right? …I don’t really know how else you would do it. I’m sure you can make it happen.”

Nearly all providers knew abortion was not offered in their hospital, yet few knew where or how to refer patients for abortion and postabortion care in the community. An ED nurse asked, “Are [abortions] available? I don’t think so… is there a clinic out there … I don’t know” (ED nurse). Confusion and misinformation about SMA legality was even more pervasive. It appeared that most providers had never considered whether SMA was illegal, though some explained they assumed it was, and were uncertain about when and to whom to report SMA cases. An ED physician who had been conflicted whether or not to report a patient said, “I wish I did know [about] laws or policies related to SMA… I don't know the actual laws about it. I mean I assumed [SMA] was illegal, because it's not under the supervision of a physician.”

Providers not only lacked formal training in abortion care but were also ill-equipped to provide comprehensive counseling and resources to patients about family planning and pregnancy options more broadly. Several providers expressed interest in becoming better trained to support the emotional needs of their patients and provide options counseling, resources, and information for those seeking family planning, adoption, abortion, or postabortion care. An ED nurse explained: “[Abortion] is not even part of our program, like, hey, if we see this, what can we give them? There’s not a doctor you can refer them to. … There’s nothing for them. You know, and it’s sad, too, because it’s like a 20-year-old who comes and doesn’t want to have a child, and now you hear people say, ‘Well, she shouldn’t have had sex.’ …And I feel like that’s terrible.”

A few providers acknowledged that it would be helpful to learn about SMA in particular, even if they do not plan to be involved in abortion care directly. For example, an L&D physician said: “I feel that we need more training. I feel that even if you’re not going to provide abortion services… that I should be prepared to talk to my patients about their different options and be able to counsel my patients objectively.” Similarly, an ED nurse said there is a gap in training on support services for SMA patients and in understanding how their counseling needs may differ from those of patients undergoing a spontaneous miscarriage. She explained: “You know, we get a lot of training about bereavement and if she comes in with like a fetal demise or a pregnancy loss to provide comfort and that kind of side of it. But we don't really get anything more specific to a self-terminated pregnancy. …Kind of like a different set of training but around a similar—around pregnancy loss.”

Several providers reflected on whether it was within the scope of work of an ED provider to counsel SMA patients, implying that the ED provider’s primary responsibility is typically to meet the acute medical needs of the patient. In general, providers said they did not need additional training on medical management of incomplete abortion or more serious complications related to pregnancy loss in order to adequately meet the needs of their patients. A physician who worked in the ED said, “…I don’t know if it’s necessarily the role of the ER [to counsel patients]. As for the medical aspect of it, we’re absolutely equipped for it—we do it every day. But the social aspect of it—we’re not really equipped.” Related to this, a few providers recommended better referral systems between the ED and L&D hospital units for patients who have miscarried or attempted SMA. This sentiment was echoed by both L&D and ED providers. An L&D physician commented: “I actually was hoping that up in L&D, where we exclusively see nothing but pregnant patients, that perhaps we can also see all pregnant patients, regardless of their gestation, up in L&D. I believe that would be a better environment for the patient. There's L&D nurses that are well trained in vaginal exams and … I believe maybe we're just in that better mindset to deal with the emotional and the psychological issues that come with a pregnant patient.” An ED nurse similarly explained: “… that would be really nice to have, even if someone from L&D came down and talked to [the patients]. …I would love to have them more involved with us in these sort of cases.”

## Discussion

These hospital provider experiences give insights into the presentation and clinical course of patients attempting SMA who obtained care at hospitals along the Texas-Mexico border and how decisions are made regarding their care. Overall, respondents reported seeing SMA cases in the hospital, but not necessarily in increasing numbers. Most providers agreed that SMA patients should receive the same care as any patient experiencing pregnancy loss, but they had less certainty about reporting. This study supports prior quantitative findings, which indicate that SMA patients seek hospital care and that most abortion-related ED visits require only observation care without treatment [14, 15]. Ralph et al. found that less than one-third of a large nationally representative sample of women ages 18–49 reported that their SMA method worked to end the pregnancy, 11.4% reported miscarrying later in pregnancy, and a total of 11% reported experiencing a complication requiring treatment by a physician or nurse. Our findings related to uncertainty around SMA reporting and a lack of provider knowledge about abortion legality and resources point to opportunities for improved hospital provider training to ensure that SMA patients get the care they need. This is supported by prior research on the challenges of providing miscarriage care in the ED, including the similarity between abortion and miscarriage management and limited capacity to address emotional aspects of the care [17]. Perspectives on treatment of SMA in the hospital setting are particularly useful given restrictions that may limit access to facility-based abortion care in the border region and beyond.

Hospital providers in the border region appeared to be equipped to meet the clinical needs of SMA patients, given that SMA patients often have similar clinical presentations as patients with pregnancy loss [22, 23]. But appropriate comprehensive care for SMA patients in the hospital setting also requires that providers be able to manage SMA with medications, to identify when use of expectant management is reasonable, to recognize potential complications of unsafe methods, to provide relevant counseling and information, and to limit or reduce patients’ risk of criminalization [23]. With increased access to mifepristone and misoprostol through informal markets and less use of dangerous SMA methods, the potential clinical risks associated with SMA are likely to be low. Though evidence is limited, studies show that people can use medication abortion safely on their own with minimal supervision [23–25].

Comprehensive and compassionate postabortion care is also important in the hospital setting for the treatment of patients following an SMA attempt. The five essential elements of quality postabortion care, as recommended by The World Health Organization, include: (1) prevention of unsafe abortion through community and service provider partnerships, (2) timely identification and response to women’s emotional and physical health needs, (3) treatment of incomplete and unsafe abortion and complications, (4) provision of family planning and contraceptive services, and (5) referral to reproductive and other health services as needed [26]. According to Dickens et al. 2019, it is not only appropriate to apply postabortion care to the context of SMA, it is part of the professional, ethical and legal duties of all providers [27]. Importantly, the World Health Organization framework does not take into account the risk of criminalization faced by patients in the US [23]. Based on the present study’s findings, two essential elements of postabortion care may be in need of improvement in the hospital setting in particular. These include: timely identification and response to women’s emotional and physical health needs and referral to reproductive and other health services as needed [26]. Similar to findings from Dennis et al. on miscarriage management in the ED [17], most hospital providers in this study were not equipped to meet the emotional as well as physical needs of their patients in the context of SMA, including being aware of and sensitive to abortion stigma and potential legal risks to the patient of reporting SMA. In addition, this study revealed that the presence of abortion stigma in the hospital setting was perpetuated by some providers, which could lead to diminished care quality. Particularly in the border region, some patient characteristics, such as immigration status and income level, may put patient health and wellbeing at additional risk if police are unnecessarily involved. Many providers lacked awareness of the availability of abortion services in the region and were unprepared to meet the counseling needs specific to SMA patients, including providing accurate referral resources and information about the legality of SMA and abortion. This may be partially explained by the fact that nurses comprised the majority of the sample, and nurses tend to have lower levels of knowledge about clinical and legal aspects of abortion relative to their physician counterparts [28].

Findings from the present study suggest that providers, particularly those who are unaware of abortion and SMA legality, may report potential SMA cases to the police unnecessarily, perhaps due to concerns about liability in the context of a highly litigious medical system. Care can usually be given without knowledge of whether the abortion is self-managed or spontaneous [23] and, in the case that one does learn of a patient’s SMA attempt, no state currently requires healthcare providers to report SMA, even when the patient is a minor. Hospital clinicians must be aware of the potential risk to patients’ legal safety of involving CPS or police in suspected SMA cases. The involvement of police in cases of pregnancy complications, including miscarriage, can result in the detention and prosecution of individuals [29]. Laws or practices that criminalize SMA may put patients experiencing pregnancy complications (related or unrelated to SMA) at medical risk by discouraging them from seeking care when they need it [30]. Reporting is also problematic because caregivers are more likely to report patients of color and low-income patients than white or affluent patients in similar circumstances [31]. Leading national medical organizations oppose the prosecution and punishment of pregnant women who use substances [32–34], including for the purposes of SMA [35]. The American College of Obstetricians and Gynecologists (ACOG) has explicitly stated that forcing physicians to share information about their patients, including related to a potential SMA attempt, jeopardizes the patient-provider relationship [35, 36].

This study has several limitations. Participants discussed SMA cases that occurred at any point in their hospital career and therefore were subject to recall bias. It is possible that providers were more likely to remember the SMA cases that required intervention relative to those that required only routine miscarriage management. In order to address this, we have only included in the analysis the cases for which providers stated or implied that they were involved directly in the patient’s care. Another limitation is that we did not interview social workers at the hospitals who were involved in the decision to include authorities in SMA cases, so we are not able to present their direct experiences or decision-making protocols except through the perspectives of providers. Provider perspectives on SMA may vary by provider type, hospital, and hospital department; however, this study was not designed to investigate these subgroup differences. Finally, these data cannot be used to estimate the prevalence of SMA, treatment methods, or complications at these sites, or to represent the important perspectives of patients or providers outside of the border region—all of which are aims that were outside of the scope of this study. In future research, quantitative data are needed to understand SMA trends, prevalence of SMA complications, and prevalence of hospital treatment for SMA complications. Despite its limitations, this is one of the first studies to investigate hospital provider perspectives on SMA.

The qualitative findings presented here contribute to a nuanced understanding of the range of types of SMA cases that hospital providers along the Texas-Mexico border encounter and the treatment they provide. While the findings are not generalizable beyond Texas, or the Texas-Mexico border region specifically, facility-based abortion care is being restricted in many states across the US, which may prompt more patients to attempt SMA. Because people who attempt SMA, and those suspected of having attempted SMA, have been criminalized, it is important that hospital-based providers have appropriate training and preparation on the clinical care and legal protections these patients require.

## Data Availability

The datasets generated and/or analyzed during the current study are not publicly available due to privacy and confidentiality concerns but are available from the corresponding author on reasonable request.

## Abbreviations

APC: Advanced Practice Clinician
ED: Emergency Department
L&D: Labor & Delivery
SMA: Self-managed abortion
CPS: Child Protective Services
